# Evaluation of a community-based aetiological approach for sexually transmitted infections management for youth in Zimbabwe: intervention findings from the STICH cluster randomised trial

**DOI:** 10.1016/j.eclinm.2023.102125

**Published:** 2023-08-03

**Authors:** Chido Dziva Chikwari, Victoria Simms, Katharina Kranzer, Ethel Dauya, Tsitsi Bandason, Mandikudza Tembo, Constancia Mavodza, Anna Machiha, Owen Mugurungi, Primrose Musiyandaka, Tinashe Mwaturura, Nkazimulo Tshuma, Sarah Bernays, Constance Mackworth-Young, Joanna Busza, Suzanna C. Francis, Richard J. Hayes, Rashida A. Ferrand

**Affiliations:** a MRC International Statistics & Epidemiology Group, Department of Infectious Disease Epidemiology, London School of Hygiene & Tropical Medicine, London, UK; b Biomedical Research and Training Institute, Harare, Zimbabwe; c Clinical Research Department, London School of Hygiene & Tropical Medicine, London, UK; d Division of Infectious Diseases and Tropical Medicine, University Hospital, LMU Munich, Munich, Germany; e AIDS and TB Unit, Ministry of Health and Child Care, Harare, Zimbabwe; f AIDS Healthcare Foundation, Zimbabwe; g School of Public Health, University of Sydney, Sydney, Australia; h Department of Global Health and Development, London School of Hygiene & Tropical Medicine, London, UK; i Department of Public Health, Environments and Society, London School of Hygiene & Tropical Medicine, London, UK

**Keywords:** STI testing, Youth, HIV, Integration

## Abstract

**Background:**

Young people are at high risk of sexually transmitted infections (STIs). We report STI testing uptake, prevalence and incidence within a community-based integrated HIV and sexual and reproductive health service for youth, being evaluated in a cluster randomised trial in Zimbabwe.

**Methods:**

This paper reports the intervention findings of the cluster randomised trial whereby STI testing was offered to all service attendees (16–24 years) in 12 intervention clusters over 12 months between October 5, 2020, and December 17, 2021, in Zimbabwe. Testing for *Chlamydia trachomatis* [CT] and *Neisseria gonorrhoeae* [NG] was offered to males and females with results available in one week and follow-up of test-positive clients by telephone. *Trichomonas vaginalis* [TV] testing was offered to females only with same day results and treatment. Youth testing positive for any STI were offered partner notification slips and free treatment for partners. This trial was registered with ISRCTN Registry, ISRCTN15013425.

**Findings:**

Overall, 8549/9891 (86.1%) eligible youth accepted CT/NG testing. Prevalence of CT and NG was 14.7% (95% CI 13.6–15.8) and 2.8% (95% CI 2.2–3.6) respectively. Combined prevalence of CT, NG or TV in women was 23.2% (95% CI 21.5–25.0). After adjusting for cluster, age and sex, the odds of NG were increased in those living with HIV (aOR 3.14, 95% CI 2.21–4.47). The incidence rate among those who initially tested negative for CT or NG was 25.6/100PY (95% CI 20.6–31.8). CT/NG treatment uptake was 924/1526 (60.6%). TV treatment uptake was 483/489 (98.8%). A partner returned for treatment for 103/1807 clients (5.7%).

**Interpretation:**

Our findings show high acceptability of STI testing among youth. STI prevalence was high particularly among females and youth with HIV, underscoring the need for integration of HIV and STI services.

**Funding:**

10.13039/501100000265MRC/10.13039/501100000269ESRC/10.13039/501100000278DFID/10.13039/501100000272NIHR (MR/T040327/1) and 10.13039/100010269Wellcome Trust (206316/Z/17/Z).

**Research in context**

**Evidence before this study**

Young people aged 10–24 years are at high risk of HIV and STIs. On March 15th, 2023, we searched MEDLINE for studies evaluating diagnostic STI testing strategies for adolescents and young people in Africa without restrictions on date or language. Using the search terms “STI testing”, “Africa”, “adolescents”, “youth” and “young people”. The search identified 272 full text publications and their abstracts were reviewed to assess relevance. The full text for 28 publications were reviewed and among them six were relevant to our question; two publications were pilot studies of our study in Zimbabwe, one was an evaluation nested with schools in South Africa, one was an evaluation among pregnant women in Botswana and another was among young people living with HIV in Uganda. Besides our study no community based STI testing interventions for youth were found despite the high burden.

**Added value of this study**

Our study reports high uptake of STI testing (86%) and high STI prevalence in a community-based setting among youth. Gaps in knowledge about STI testing and burden among youth were in the past largely due to the unavailability of cheap, accurate and simple diagnostics for STIs. Our study provides critical evidence to address this gap.

**Implications of all the available evidence**

This study provides strong evidence for recommending a shift away from syndromic management which has low sensitivity and specificity to aetiological approaches ideally incorporating point-of-care diagnostics that will facilitate timely treatment uptake among high-risk groups such as youth.

## Introduction

The global prevalence of sexually transmitted infections (STIs) remains high with more than one million infections per day of curable STIs (syphilis, gonorrhoea, chlamydia and trichomoniasis) in 2020.^1^ Notably, from 1990 to 2019 the sub-Saharan African (SSA) region had the highest age standardised incidence rate of STIs of any global region.^2^ Untreated, STIs lead to long-term morbidity including pelvic inflammatory disease, infertility, adverse pregnancy outcomes and neonatal death. STIs are also associated with significantly increased risk of HIV transmission but, in recent years, STI control has been neglected as an HIV prevention strategy.^1^^,^^3^ STI control in resource-constrained settings has relied on syndromic management, due to limited laboratory infrastructure.^4^ This is problematic as >70% of curable STIs are asymptomatic and thus not identified or treated, facilitating continued transmission and impeding STI control.^4^

The availability of sensitive and specific assays that are user-friendly e.g., through use of molecular closed system platforms or that require no equipment such as lateral flow tests, now make it possible to shift away from syndromic to aetiological approaches for STI control.^1^^,^^4^ However, there is sparse evidence on *how* to implement such approaches to maximise effectiveness. The WHO 2022–2030 global health sector strategy on HIV, viral hepatitis and STIs calls for integration of STI and HIV responses and a focus on populations most at risk of STIs.^1^

Adolescents and young people aged 10–24 years are at high risk of HIV and STIs.^2^ Multiple health system factors such as vertical programmes and lack of age-appropriate services or health worker training, compounded by sociocultural factors resulting in stigmatisation, impede young people's access to and engagement with sexual and reproductive health (SRH) services available through existing health facilities.^5^^,^^6^ Addressing these barriers will be critically important if aetiological approaches to STI testing are to be effectively implemented in this high-risk group.

We conducted a cluster randomised trial to evaluate the impact of community-based provision of STI testing and management integrated within HIV and sexual and reproductive health (SRH) services on population-level STI prevalence among youth (STICH) in Zimbabwe. Health facility usage rates among youth are low globally due to substantial personal, social, legal and structural barriers, and we hypothesized that service delivery in community-based settings would improve access.^7^ Integration with HIV and SRH services may improve service engagement and uptake and improve overall programmatic efficiency.^7^ In this paper we report the uptake of testing, prevalence and incidence (among those who tested multiple times) of STIs, and completion of treatment for indexes and their partners in the intervention clusters. The impact of the intervention on population prevalence of STIs is reported elsewhere.

## Methods

### Study design and participants

The STICH trial (Trial Registration Number: ISRCTN15013425) was nested within a parent cluster randomised trial (CHIEDZA) (Trial Registration Number: NCT03719521) conducted in three provinces across Zimbabwe, each province randomised 4:4 to control (12 total) (existing health services) or intervention (12 total) clusters. A cluster was defined as a geographically demarcated area with a multi-purpose community centre from where weekly integrated HIV and SRH services as well as general health counselling were delivered to intervention cluster residents aged 16–24 years at the identified community centre in each intervention cluster. The design of the intervention was informed by formative work conducted with young people and is described elsewhere.^8^ The detailed intervention protocol is described elsewhere.^9^ CHIEDZA services included HIV testing, HIV treatment and adherence support for those living with HIV, contraception, menstrual health, syndromic management of STIs, risk reduction and general health counselling offered by a multidisciplinary team of service providers over 30 months. The start of the intervention period was staggered, with Harare starting in April 2019, followed three and six months later by the introduction of the intervention in Bulawayo and Mashonaland East province respectively. Youth mobilisers were responsible for sensitising the community and other young people about CHIEDZA in each intervention cluster.^9^ All services were voluntary and offered free of cost. The nested STI trial (“STICH”: *STIs in CHIEDZA*) was conducted in two of the three CHIEDZA provinces (Harare and Bulawayo). This paper reports findings from the eight STICH intervention clusters in Harare and Bulawayo.

The trial methodology and randomisation have been described previously.^9^ Testing for STIs (described below) was offered universally to all CHIEDZA service attendees, regardless of whether they reported symptoms, in the final 12 months of the 30 month CHIEDZA intervention (5 October 2020–30 September 2021 in Harare; 4 January 2021–17 December 2021 in Bulawayo). To improve engagement and minimise barriers to service uptake clients were not asked about their sexual behaviour. Those who presented with symptoms were managed as per national guidelines for syndromic management but were also offered STI testing.^10^ Clients could access re-testing after three months following a test, and sooner if they had new or persisting symptoms. Youth who tested positive for any STI were actively encouraged to retest after three months.

### STI testing

Testing for *Chlamydia trachomatis* [CT] and *Neisseria gonorrhoeae* [NG] using urine samples was offered to both males and females with results available in one week. Testing for *Trichomonas vaginalis* [TV] using self-collected vaginal swabs was offered to females only. CT/NG testing was conducted off site at a laboratory using the GeneXpert assay (Cepheid, South Africa). TV testing was conducted on site using the point-of-care OSOM lateral flow test (Sekisui Diagnostics, USA) with results available within 15 min and same day treatment provided. The intervention was piloted prior to the trial and at this time low uptake of testing, lack of information and misconceptions about STIs among youth were observed.^11^ This was addressed through development of information leaflets that addressed the identified gaps in information which were made available for all young people in each site during the intervention and a once off re-training of the providers.

Any client testing positive for CT/NG was followed up via telephone within 6 days of the test and at 2, 4 8- and 12-weeks following testing if not contactable and asked to return to the site for their results. A pseudonym provided at the time of testing was used to confirm identity when the client was contacted. A pseudonym was used to protect the anonymity of the client and no results were provided over the phone. Those with negative test results were not contacted. Treatment was provided as per national guidelines.^10^ Anyone testing positive for any STI was offered partner notification (PN) slips and their partners were encouraged to access the service for treatment free of cost regardless of their age or whether they lived in the intervention cluster.

Given that STICH was embedded within the CHIEDZA trial, all CHIEDZA services including HIV testing and care and SRH services were offered alongside STI testing to attendees.

### Outcomes

The primary outcome for this study was the uptake of STI testing, defined as the number of eligible youth attending the CHIEDZA service during the 12 months when STI testing was offered who accepted STI testing. Secondary outcomes were the prevalence of STIs defined as youth testing positive for an STI among those who received an STI test during the intervention period, and treatment uptake defined as the number of youth taking up STI treatment among those who tested positive for an STI. We also investigated incidence among clients who underwent STI testing on more than one occasion. Outcomes for CT/NG (males and females) and TV (females only) are reported separately. Uptake of partner treatment was also investigated and defined as the number of partners taking up treatment at CHIEDZA among those who tested positive for any STI.

### Data management and statistical analysis

Each client's attendance was recorded using biometrics. A fingerprint record was taken at each visit which was converted to a Global Unique Identification number (GUID) using SIMPRINTS software (Cambridge, UK). Data on age, sex, type of STI test and other services taken up at each visit were recorded on electronic tablets using SurveyCTO software and linked to the GUID. These enabled services taken and repeated STI testing to be tracked over time.

Analyses were performed using STATA version 17.0 (StataCorp, Texas, USA). Continuous variables were summarised as means and SDs or medians and IQRs, and categorical variables as counts and percentages. The prevalence of STIs was reported stratified by age, sex, HIV status and province, adjusted for within-cluster correlation. Using logistic regression, we also evaluated the association of HIV status with STIs adjusting for age, sex, and cluster. Among clients who tested negative and were then retested at a later visit, the incidence rate of STIs was estimated per 100 person-years (PY) of follow-up calculated as time between previous test and retest.

Ethical approval for the CHIEDZA and STICH trials was obtained from the Medical Research Council of Zimbabwe [MRCZ/A/2387], the Biomedical Research and Training Institute Institutional Review Board [AP149/2018] and the London School of Hygiene & Tropical Medicine Ethics Committee [16124/RR/11602]. All intervention attendees provided verbal consent for services including STI testing. The requirement for guardian consent to access services for 16–18-year-olds was waived by the ethics committees.

### Role of funding source

The funders of the study had no role in study design, data collection, data analysis, data interpretation, writing of the report, or the decision to submit the study for publication. All authors had full access to the study data and the corresponding author had final responsibility for the publication.

## Results

### Uptake of STI testing

Over the 12-month intervention period, 10,691 clients (71.2% female) attended CHIEDZA services in the eight intervention clusters in Harare and Bulawayo. There was a stockout of test kits between April–May 2020 and in October 2021, caused by delayed importation during the COVID-19 pandemic.

**Fig. 1:**
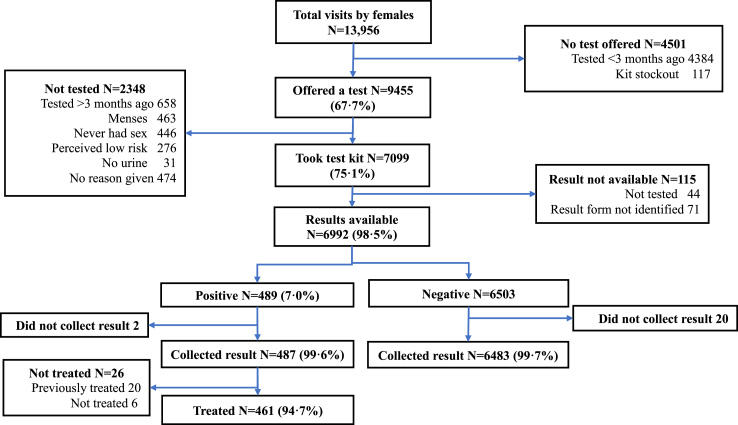
**TV Flowchart.** Client flowchart for Trichomonas vaginalis [TV] testing among females showing testing and treatment uptake.

Excluding visits that occurred when there was a stockout of test kits and those where the client was not eligible for STI testing (e.g., having tested within the previous 3 months), 9891 unique eligible clients attended the service over the intervention period. Of these, 8549 (86.1%) clients accepted CT/NG testing (Table 1). The proportion of clients who accepted CT/NG testing was higher in Harare (91.7%) than in Bulawayo (80.7%). Uptake of STI testing did not vary by sex, but increased with age, from 78.9% among 16–18-year-olds to 91.6% in those aged >21 years. Most participants who accepted a test did so on their first visit (8213/8549, 96.1%). Of the 7501 eligible women, 6388 (85.2%) ever accepted a TV test (Fig. 1). As for CT/NG, TV test uptake was higher in Harare (90.0%) than Bulawayo (79.8%) and increased with age from 75.4% in adolescents aged 16–18 years to 92.0% in those aged >21 years. There was no difference in CT/NG testing uptake between those with known HIV positive status (92.9% uptake) and those with known HIV negative status (88.4% uptake), but those who did not know their HIV status were less likely to accept a CT/NG test (43.1% uptake) (Table 1). Overall, the main reasons for non-uptake of STI testing were having been tested over three months ago and declining a second test, not having had sexual debut and currently menstruating.

**Table 1:** Uptake of CT/NG and TV testing by individual.

Variable	Category	Total			Females		
		Eligible N	Ever had a CT or NG test, n (%)	Chi^2^, p	Eligible N	Ever had a TV test, n (%)	Chi^2^, p
N		9891	8549 (86.4)		7501	6388 (85.2)	
Age	16–18	3414	2694 (78.9)	258.5, <0.001	2670	2012 (75.4)	322.4, <0.001
	19–21	3485	3114 (89.4)		2512	2243 (89.3)	
	22–27	2992	2741 (91.6)		2319	2133 (92.0)	
Sex	Male	2825	2471 (87.5)	3.6, 0.057			
	Female	7066	6078 (86.0)				
Province	Harare	5127	4703 (91.7)	254.8, <0.001	3969	3570 (90.0)	152.7, <0.001
	Bulawayo YY	4764	3846 (80.7)		3532	2818 (79.8)	
Total number of visits during STICH intervention	1	5598	4481 (80.1)	453.3, <0.001	4469	3508 (78.5)	389.4, <0.001
	2–5	3858	3641 (94.4)		2786	2642 (94.8)	
	>5	435	427 (98.2)		246	238 (96.8)	
HIV status	Positive	477	443 (92.9)	814.5, <0.001	441	392 (88.9)	574.5, <0.001
	Negative	8934	7899 (88.4)		6714	5856 (87.2)	
	Unknown	480	207 (43.1)		346	140 (40.5)	
Visit when first accepted STI testing (during STICH intervention)	Never	1342			1114		
	First visit	8213	8213		6132	6132	
	Second visit	293	293		226	226	
	Third or later visit	43	43		30	30

### Prevalence and incidence of STIs

The prevalence of CT and NG was 14.7% (95% CI 13.6–15.8) and 2.8% (95% CI 2.2–3.6) respectively (Table 2). The prevalence of CT and NG was twice as high in females than males. The combined prevalence of CT, NG or TV in women was 23.2% (95% CI 21.5–25.0). In men, who were only tested for CT and NG, the combined prevalence of the two STIs was 9.8% (95% CI 8.2–11.8).

**Table 2:** Prevalence of STIs.

Variable	Category	N	CT		NG		Either CT or NG		TV (Females only)		
			N cases	% (95% CI)	N cases	% (95% CI)	N cases	% (95% CI)	N female	N cases	% (95% CI)
Total		9368	1376	14.7 (13.6–15.8)	264	2.8 (2.2–3.6)	1526	16.3 (15.0–17.6)	6992	489	7.0 (6.3–7.8)
Age (years)	16–18	2827	239	8.5 (6.2–11.5)	52	1.8 (1.3–2.7)	267	9.4 (7.1–12.5)	2097	102	4.9 (3.8–6.1)
	19–21	3438	572	16.6 (15.7–17.6)	103	3.0 (2.1–4.2)	631	18.4 (16.8–20.0)	2491	187	7.5 (6.3–9.0)
	22–27	3103	565	18.2 (16.6–19.9)	109	3.5 (2.7–4.5)	628	20.2 (18.5–22.0)	2404	200	8.3 (7.0–9.8)
Sex	Male	2755	241	8.7 (7.4–10.4)	46	1.7 (1.0–2.8)	271	9.8 (8.2–11.8)			–
	Female	6613	1135	17.2 (15.6–18.9)	218	3.3 (2.7–4.0)	1255	19.0 (17.3–20.8)	6992	489	7.0 (6.3–7.8)
Province	Harare	4799	652	13.6 (13.0–14.1)	122	2.5 (1.8–3.6)	726	15.1 (14.3–16.0)	3613	263	7.3 (6.3–8.4)
	Bulawayo	4569	724	15.8 (15.2–16.5)	142	3.1 (2.3–4.1)	800	17.5 (16.2–18.9)	3379	226	6.7 (5.7–7.8)
HIV status	Positive	470	70	14.9 (11.6–18.9)	42	8.9 (6.3–12.5)	98	20.9 (16.9–25.4)	414	60	14.5 (11.4–18.2)
	Negative	8428	1255	14.9 (13.7–16.2)	216	2.6 (2.0–3.2)	1372	16.3 (14.9–17.8)	6261	412	6.6 (5.8–7.5)
	Unknown	470	51	10.8 (7.2–16.0)	6	1.3 (0.3–4.9)	56	11.9 (8.5–16.4)	317	17	5.4 (3.4–8.4)

There was no difference in prevalence of CT by HIV status, but NG was more prevalent among people living with HIV (8.9%; 95% CI 6.3–12.5) than those who were HIV negative (2.6%; 95% CI 2.0–3.2). After adjusting for cluster, age and sex, the odds of NG (aOR 3.14, 95% CI 2.21–4.47) but not CT (aOR 0.79, 95% CI 0.60–1.03) were increased in those who were living with HIV. Females with HIV also had higher odds of having TV than HIV-negative females (aOR 2.22, 95% CI 1.66–2.98) after adjusting for cluster and age. Increasing age was associated with increased prevalence of all three STIs, and prevalence of CT was higher in Bulawayo than in Harare.

In total, 652 participants had at least one repeat visit and CT test following a first negative CT test, enabling observation of 325.0 person-years (PY) at risk, 109.8 in men and 215.2 in women (Table 3). The incidence of CT in this group was 21.5/100 PY overall (95% CI 17.0–27.2). The incidence rate of NG was 8.4/100PY (95% CI 5.9–11.9) and the incidence rate of either CT or NG was 25.6/100PY (95% CI 20.6–31.8). For TV, 573 women had a repeat visit and TV test following a first negative TV test, representing 285.2 PY at risk, and 23 observed infections (TV incidence 8.1/100PY, 95% CI 5.4–12.1).

**Table 3:** STI incidence following a negative test result.

STI	Total			Male			Female		
	PY^a^ at risk	Positive tests	IR^b^/100PY (95% CI),	PY at risk	Positive tests	IR (95% CI), 100PY	PY at risk	Positive tests	IR (95% CI), 100PY
CT	325.0	70	21.5 (17.0–27.2)	109.8	21	19.1 (12.5–29.3)	215.2	49	22.8 (17.2–30.1)
NG	381.4	32	8.4 (5.9–11.9)	117.5	8	6.8 (3.4–13.6)	263.9	24	9.1 (6.1–13.6)
CT or NG	316.9	81	25.6 (20.6–31.8)	108.8	25	23.0 (15.5–34.0)	208.1	56	26.9 (20.7–35.0)
TV							285.2	23	8.1 (5.4–12.1)
CT, NG, or TV	314.9	84	26.7 (21.5–33.0)				206.1	59	28.6 (22.2–36.9)

a PY: Person Years at risk.

b IR: Incidence rate.

### STI treatment cascade

**Fig. 2:**
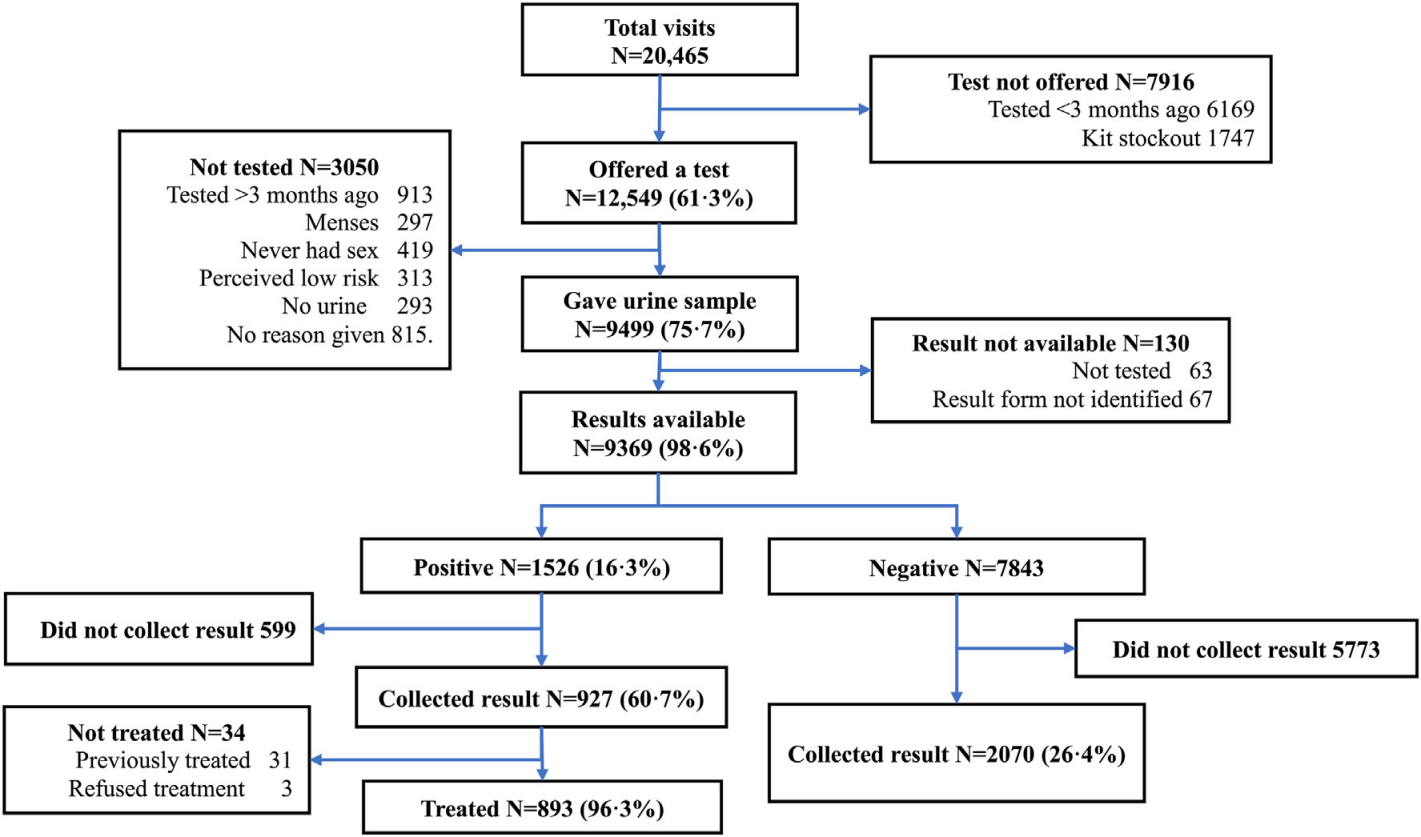
**CT/NG flowchart.** Client flowchart for *Chlamydia trachomatis* [CT] and *Neisseria gonorrhoeae* [NG] testing among males and females showing testing and treatment uptake.

The STI treatment cascade for CT/NG and TV by visit level is shown in Figs. 1 and 2. A total of 927 (60.7%) of 1526 clients with a positive CT/NG came to the site to collect their result and among them only three refused treatment. Among those who came back to the site, 31 had already been treated as part of syndromic management. Out of the 7843 participants with negative tests, 26.4% came to the site and collected their results.

Provision of TV results and treatment was almost universal (461/469 (98.3%)) given that a point-of-care test was used. Including the 20 cases that were previously treated through syndromic management, the overall treatment uptake was 481/489 (98.4%).

In total 1807 clients tested positive for at least one of the 3 STIs (CT/NG or TV) and were offered partner notification slips. Only for 103 (5.7%) did a partner return for treatment.

### Syndromic management

During the STICH intervention period, 262 clients presenting to CHIEDZA services had symptoms and underwent syndromic management of STIs. Of the 262, 67 (25.6%) had tested within the previous 3 months (16/50 (32%) had been positive at the previous test, 17 results were unknown) and 36 (13.7%) came during a stockout period, so 159 (60.7%) were offered CT/NG testing and 144 (90.6%) of those offered accepted. The prevalence of CT/NG among the symptomatic clients was 41.7% (60/144) and the negative predictive value of syndromic management was 84.1%. In all clients who tested, syndromic management had a sensitivity and specificity of 3.9% and 98.9% respectively compared to CT/NG testing. Among females only, syndromic management compared to CT, NG or TV testing had a positive predictive value of 40.0% (34/85 women with syndromic management tested positive), negative predictive value of 77.0%, sensitivity of 2.2% and specificity of 99.0%. Among males only, syndromic management compared to CT and/or NG had a sensitivity of 12.6%, specificity of 99.0%, positive predictive value of 57.6% and negative predictive value of 91.2%.

## Discussion

Our study showed high acceptability and uptake of STI testing among youth who attended the CHIEDZA community-based service, with over 85% taking up STI testing delivered as part of an integrated package of HIV and SRH services. The high uptake in our study contrasts significantly with the low uptake we observed in the pilot phase where only a third of youth took up testing.^11^ This difference may be due to refresher and additional training given to providers addressing the information gaps identified in the pilot, and adapted messaging that tackled some of the misinformation about STI testing among youth, for example HIV testing was perceived to cover “all STIs”. The revised information outlined the differences between HIV and other STIs to youth attending the service.^11^

In contrast to our study, STI testing uptake among youth in other community-based settings has generally been low. For example, among youth attending technical colleges in London, STI testing uptake was only 13%. Uptake of internet accessed STI testing among youth (16–30 years) recruited in community settings, also in London, was only 44% while uptake of facility-based testing in the same study was 24%.^12^^,^^13^ The high uptake of STI testing observed in our study may have been due to the confidential nature of services and anonymised testing routinely offered as part of an integrated package of services. No identifiable information (such as name or address) was collected, and for follow-up purposes clients were asked to provide a pseudonym. Providers were specifically trained to be non-judgemental and maintain confidentiality, and STI testing was embedded within an integrated package of broader health services offered in a community-based setting (multi-purpose community halls) which enabled young people to take up STI testing without fear of stigma from their peers, healthcare providers or communities. The uptake of STI testing out of those who came to CHIEDZA was similar in men and women. As such STI testing may be a potential strategy to improve male engagement with SRH services.^14^^,^^15^

We observed a high STI prevalence among youth with almost one in four young women (23%) testing positive for either CT/NG/TV and 10% prevalence of CT/NG among males. Only a small proportion of clients who tested positive for an STI had symptoms and were therefore treated using syndromic management. As shown by previous research, this study highlights the limitations of syndromic management, which remains the standard of care in Zimbabwe and other resource-constrained settings despite World Health Organization recommendations to move towards aetiological approaches for STI management.^16^ There were marked differences in STI prevalence by sex, with disproportionately higher prevalence of CT and NG among females. High STI risk among adolescent girls and young women is well documented, due to a range of socio-behavioural and biological factors including lower production of cervical mucous, having older sexual partners and reduced agency to negotiate safe sex.^17^, ^18^, ^19^ We observed a high incidence of STIs likely due to the high prevalence of STIs and reflective of high-risk sexual behaviour among youth. It is possible that the STI incidence in this study may also be an over-estimate of population incidence as those at higher risk may have been more likely to return to CHIEDZA within 12 months and access repeat testing.

We observed a significantly higher NG prevalence among youth living with HIV than in those who were HIV negative (8.9% vs 2.6%), and women living with HIV were more than twice as likely to have TV infection than those who were HIV negative. There was no difference by sex observed for CT. The association was cross-sectional and either those with NG may be at higher risk of acquiring HIV infection and/or those with HIV may be at higher risk of acquiring NG. This calls for integrated and bidirectional delivery of testing for HIV and other STIs, and strongly aligns to the WHO global health sector strategies on, respectively, HIV, viral hepatitis, and STIs for the period 2022–2030.^20^ A possible avenue is provision of STI testing and treatment within HIV care services, taking advantage of frequent contact with health facilities among individuals living with HIV. Similarly, individuals diagnosed with curable STIs should also be offered HIV testing.

Near universal treatment was achieved for TV, identified using a point-of-care test, in contrast to 60% for CT/NG where results were not available on the day of testing. To improve treatment uptake, STI programmes need to consider implementation of point-of-care tests to avoid missed opportunities for treatment once a diagnosis has been made.

Comprehensive STI management includes the reduction of risk of re-infection through partner treatment. We observed only 5.7% uptake of partner treatment at our sites and high rates of infection among youth who were tested more than once. Partner notification may inadvertently have been negatively impacted by the intervention design which was tailored for youth. The intervention was delivered once weekly, which reduced the opportunities for partners to present for treatment and did not collect data on partner treatment taken up at facilities which were not part of the study.^21^ Overall, partner notification has the potential for social harms which include shame, stigma, potential relationship breakdown and intimate partner violence when disclosing STI results to partners, particularly casual partners.^22^ Age-disparate sexual relationships between adolescent girls and young women and older men are common, but older men may have been reluctant to engage with youth services. When compared to other population groups these challenges with partner notification can be heightened for young people and it is therefore critical to further evaluate current challenges with partner notification for youth and identify potential strategies that could work for this age-group such as provider facilitated partner notification or social network testing.^23^ Additionally given the high prevalence of STIs, high uptake of testing and low success of partner notification among youth, rolling out aetiological testing is another way to reach partners^,^ which may have fewer social and relational barriers.

The strengths of this study were a large sample size, detailed data on the entire cascade from testing to completion of treatment and data on incidence and re-infection, albeit in a self-selected sample. We acknowledge several limitations. Our study did not collect data on sexual risk behaviours nor delineate cis-gender youth who may be represented in our study population and could have elevated risk of STIs. In addition, clients who attended the services as well as those who took up STI testing may not be representative of the entire population of sexually active youth. As such the intervention STI prevalence and incidence may not be representative of sexually active young people at population level. For 1.3% of clients who accepted a test some results were lost, and samples not tested. While vaginal swabs were used for TV testing, CT/NG testing utilised urine samples, which is associated with lower sensitivity than for genital samples and may underestimate prevalence.^24^

We found no other publications investigating community-based STI testing strategies among youth in Africa despite the high burden reported in our and other studies.^25^^,^^26^ STIs are a risk factor for HIV, but in recent years STI testing has not been considered a priority as an HIV prevention strategy. Furthermore, investments in HIV programming including HIV testing have excluded other STIs.^27^

The gaps in knowledge about STI testing and burden among youth were in the past largely due to the unavailability of cheap, accurate and simple diagnostics for STIs.^28^ However, in recent years, simpler platforms that do not require expensive infrastructure and even point-of-care tests have become available. Cost and evidence for effective contextually appropriate implementation models have been major barriers to realising the benefits of these technological advances. Our study provides critical evidence to address this gap. We believe our findings are generalizable to other urban setting within the region. Costing data and the impact of the intervention on population prevalence of STIs will be reported separately.

In conclusion our study shows high acceptability and uptake of STI testing offered as part an integrated community-based HIV and SRH service. STI prevalence among youth in this study was high, particularly so among females and youth living with HIV, underscoring the need for integration of HIV and STI services, including prevention, testing and management. We recommend a move away from syndromic management with its low sensitivity to aetiological approaches, ideally incorporating point-of-care diagnostics that will facilitate high uptake of testing and high/timely treatment. Studies should evaluate appropriately tailored strategies for partner notification considering the context and the target population.

## Contributors

CDC, RAF, RH and SF conceptualised the study and designed the study methodology. RAF, KK, and RH provided project supervision. VS and TB were responsible for data curation and verification. CDC and ED were responsible for the STICH and CHIEDZA studies administration and supervision with support from MT and CM. VS and TB conducted data analysis. SB, CMY and JB were responsible for the study process evaluation and contributed to methodology for this. KK, PM, and TM oversaw laboratory testing procedures and led methodology and supervision for this. CDC, ED, MT, CM, NT, AM, and OM were responsible for project administration including training, and protocol development. All authors reviewed and approved the final manuscript. All authors had full access to the study data and the corresponding author had final responsibility for the publication.

## Data sharing statement

Individual, anonymised participant data and a data dictionary will be available through the London School of Hygiene & Tropical Medicine repository (Data Compass) at the time of publication.

## Declaration of interests

Authors declare no competing interests.

## Appendix A Supplementary data

Translate Abstract Ndebele

Translate Abstract Shona
