# Supplementary material for: Evaluation of a community-based aetiological approach for sexually transmitted infections management for youth in Zimbabwe: intervention findings from the STICH cluster randomised trial
Source: eClinicalMedicine. 2023 Aug 3;62:102125. doi: 10.1016/j.eclinm.2023.102125 (PMC10430193; doi:10.1016/j.eclinm.2023.102125)
Supplement: Translate Abstract Ndebele [file mmc1.docx]

**The following translations in Ndebele were submitted by the authors and we reproduce them as supplied. They have not been peer reviewed. Our editorial processes have only been applied to the original abstract in English, which should serve as reference for this manuscript.”**

**STICH INTERVENTION ABSTRACT**

**Imbali**

Abantu abatsha basengozini enkulu yokuthola imkhuhlane yemacansini (STIs). Sazisa ngokwamukeleka kokuhlolwa kwama-STI, ukumemetheka kwawo kanye lobunengi bawo emphakathini kwenhlanganiso ye-HIV kanye lenkonzo yezempilakahle zemacansini, kuhlolwa kuchwayisiso olwalusenziwa ezigabeni ezakhethwa okungahlelwanga eZimbabwe.

**Indlela**

Iphepha leli lazisa impumela yohlelo ezigabeni ezazingahlelwanga lapho ukuhlolwa kwe-STI kwaphiwa bonke ababekhona ehlelweni (abaleminyaka engu-16-24) ezigabeni ezingu-12 lapho okwakuqhutshwa loluhlelo phakathi kwenyanga eziyi-12 phakathi kuka-October 5, 2020, loDecember 17, 2021, eZimbabwe. Abesilisa labesifazane baphiwa ithuba lokuhlolwa i-Chlamydia trachomatis [CT] kanye le-Neisseria gonorrhoeae [NG] bephiwa impumela ngemva kweviki eyodwa kanye lokulandelelwa ngocingo kwabantu abatholakala belomkhuhlane wemacansini. Abesifazane kuphela yibo abahlolwa i-Trichomonas vaginalis [TV] impumela babeyiphiwa ngelanga lonalelo kanye lokwelatshwa. Abatsha ababetholakala bale noma yiyiphi i-STI babephiwa I partner notification slip, phezu kwalokho abangane babo babeselatshwa mahala. Loluchwayisiso lwalubhaliswe le-ISRCTN Registry,ISRCTN15013425.

**Okutholakeleyo**

Sekukonke, abatsha ababefanele abangu-8549/9891 (86·1%) bavuma ukuhlolwa i-CT/NG. Ukumemetheka kwe-CT le-NG kwakuyi 14·7% (95% CI 13·6-15·8) le-2·8% (95% CI 2·2-3·6) ngokulandelana. Ukuvama okuhlanganisiweyo okwe-CT, NG noma i-TV kwabesifazane kwabayi-23·2% (95% CI 21·5-25·0). Ngemva kokulungiswa kwezigaba, iminyaka kanye lokuthi ngumuntu owesifazane kumbe ngowesilisa yini, ithuba le-NG langezelela kulabo abaphila le-HIV (aOR 3·14, 95% CI 2·21-4·47). Ithuba lezehlakalo phakathi kwalabo abaqala bahlola batholakala ukuthi abala -CT noma i-NG bekuyi-25·6/100PY (95%CI 20·6-31·8). Abavuma ukwelatshwa i-CT/NG babengu-924/1526 (60·6%). Abavuma ukwelatshwa i-TV babengu-483/489 (98·8%). Abangane abaphenduka ukuzelatshwa babengu-103/1807 (5·7%).

**Ukutolika**
Sithole ukuthi banengi abatsha abavuma ukuhlolwa ama-STI. Ukumemetheka kwe-STI bekuphezulu kakhulu kwabesifazaneni kanye labatsha abale-HIV, kugcizelela isidingo sokuhlanganiswa kwezinsizakalo ze-HIV kanye lama-STI.
