# Supplementary material for: Evaluation of a community-based aetiological approach for sexually transmitted infections management for youth in Zimbabwe: intervention findings from the STICH cluster randomised trial
Source: eClinicalMedicine. 2023 Aug 3;62:102125. doi: 10.1016/j.eclinm.2023.102125 (PMC10430193; doi:10.1016/j.eclinm.2023.102125)
Supplement: Translate Abstract Shona [file mmc2.docx]

**The following translations in Shona were submitted by the authors and we reproduce them as supplied. They have not been peer reviewed. Our editorial processes have only been applied to the original abstract in English, which should serve as reference for this manuscript.”**

**STICH INTERVENTION ABSTRACT**

**Nhoroondo**

Vechidiki varipanjodzi yekubatira zvirwere zvepabonde (STIs). Tinozivisa huwandu

hwevakaongororwa STI, huzhinji hwevane STI uye avo vatsva vanenge vawaniwa vene STI

munharaunda paongooro dzakaitwa dzaitarisa HIV nezvehutano hwepabonde hwevechidiki

panguva imwechete, zvikaita muongororo yecluster-randomised trial muZimbabwe.

**Nzira**

Gwaro rino richapa zvakabuda pakaitwa ongororo dzeSTI kune vechidiki vose vane makore

ari pakati pegumi nematanhatu kusvika pamakumi maviri nemana (16-24 years) munzvimbo gumi nembiri dzaipa ongororo kwemwedzi inodarika gumi nemiviri (12 months) kubva musi

wa 5 October, 2020 kusvika musi wa 17 December 2021 mu Zimbabwe. Ongororo ye Chlamydia (CT) neGonorrhoea (NG) dzakaitwa kuvanhukadzi zvose nevanhurume vachipihwa maresults muvhiki imwechete uye nekuteverwa kwevawanikwa vaine maSTI aya kuchishandiswa runhare. Trichomonas (TV) yaiongororwa kuvanhukadzi chete zvekare maresults aibuda muzuvarimwe uye nekubva varapwa zuva iroro. Vechidiki vose vaiwanika vane chero mhando yeSTI vaibva vapihwa tsamba yekuti shamwari dzavo dzepabonde dziwane kurapwawo mahara. Chirongwa chino chakanyoreswa ku ISRCTN Registry, ISRCTN15013425

**Zvawanikwa**

Zvasangana, 8549/9891 (86·1%) vechidiki vaikodzera vakabvuma kuongororwa CT/NG. Huwandu hweCT ne NG hwaiva 14·7% (95% CI 13·6-15·8) ne 2·8% (95% CI 2·2-3·6) zvakatevedzana. Huwandu hweCT, NG kana TV kuvanhukadzi hwaiva 23·2% (95% CI 21·5-25·0). Mushure mekugadzirisa nharaunda, makore uye kuti munhukadzi kana munhurume, kuwanikwa kweNG kwakawedzerwa kune avo vanorarama neHIV (aOR 3·14, 95% CI 2·21-4·47). Chiitiko ichi pakati peavo vakatanga vawanikwa vasina CT kana NG yaiva 25·6/100PY (95% CI 20·6-31 ·8). Vakarapwa CT/NG vaiva 924/1526 (60·6%). Vakarapwa TV vaiva 483/489 (98·8%). Shamwari dzepabonde dzakadzoka kuzorapwa dzaiva 103/1807 (5·7%).

**Dudziro**
Zvatinowana zvinoratidza kuti kuongororwa maSTI kwakagamuchirwa zvakanyanya pakati pevechidiki. Huwandu hwemaSTI hwakanyanya kunyanya kuvanhukadzi pamwe nevechidiki vane HIV, zvichisimbisa kukosha kwekubatanidzwa kwemabasa eHIV nemaSTI.
